# Interaction of Mitochondrial Calcium and ROS in Neurodegeneration

**DOI:** 10.3390/cells11040706

**Published:** 2022-02-17

**Authors:** Artyom Y. Baev, Andrey Y. Vinokurov, Irina N. Novikova, Viktor V. Dremin, Elena V. Potapova, Andrey Y. Abramov

**Affiliations:** 1 Laboratory of Experimental Biophysics, Centre for Advanced Technologies, Tashkent 100174, Uzbekistan; baev.a.yu@gmail.com; 2 Cell Physiology and Pathology Laboratory, Orel State University, 302026 Orel, Russia; tolmach_88@mail.ru (A.Y.V.); irina.makovik@gmail.com (I.N.N.); v.dremin@oreluniver.ru (V.V.D.); e.potapova@oreluniver.ru (E.V.P.); 3 College of Engineering and Physical Sciences, Aston University, Birmingham B4 7ET, UK; 4 Department of Clinical and Movement Neurosciences, UCL Queen Square Institute of Neurology, Queen Square, London WC1N 3BG, UK

**Keywords:** neurodegeneration, mitochondria, calcium, reactive oxygen species, cell death, permeability transition pore, neuron

## Abstract

Neurodegenerative disorders are currently incurable devastating diseases which are characterized by the slow and progressive loss of neurons in specific brain regions. Progress in the investigation of the mechanisms of these disorders helped to identify a number of genes associated with familial forms of these diseases and a number of toxins and risk factors which trigger sporadic and toxic forms of these diseases. Recently, some similarities in the mechanisms of neurodegenerative diseases were identified, including the involvement of mitochondria, oxidative stress, and the abnormality of Ca^2+^ signaling in neurons and astrocytes. Thus, mitochondria produce reactive oxygen species during metabolism which play a further role in redox signaling, but this may also act as an additional trigger for abnormal mitochondrial calcium handling, resulting in mitochondrial calcium overload. Combinations of these factors can be the trigger of neuronal cell death in some pathologies. Here, we review the latest literature on the crosstalk of reactive oxygen species and Ca^2+^ in brain mitochondria in physiology and beyond, considering how changes in mitochondrial metabolism or redox signaling can convert this interaction into a pathological event.

## 1. Introduction

The central nervous system controls most of the bodily functions of higher organisms—behavior, cognition, movement, and many others. Such a variety of functions are regulated by distinguished neuro- and gliatransmitters and modulators and various receptors. Intracellular signaling in neurons and astrocytes as in most cell types is regulated by second messengers including Ca^2+^, which orchestrates almost all processes in the cell [1].

**Figure 1:**
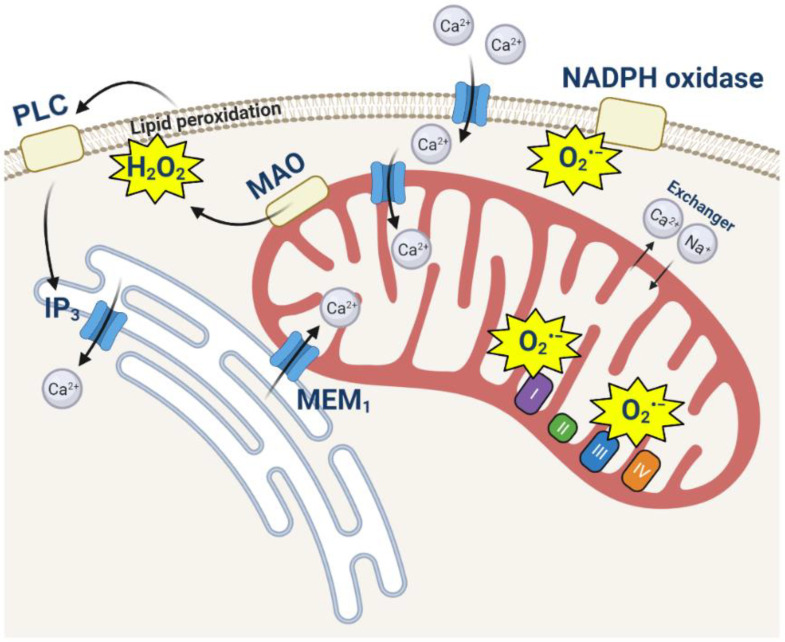
Interaction of calcium and ROS in intracellular signaling. Activation of NADPH oxidase by Ca^2+^ induces ROS production which, in combination with ROS produced in mitochondria or by MAO, can stimulate lipid peroxidation, phospholipase C (PLC) activation, and IP3 production that lead to an additional calcium signal.

Calcium signaling is a sophisticated process based on the calcium concentration gradients between cytosol and extracellular space and between cytosol and intracellular calcium stores (predominantly the endoplasmic reticulum). Mitochondria play the role of temporary calcium buffer which protects cells against high Ca^2+^ in the cytosol, which at the same time simulates the electron transport chain of mitochondria and ATP production [2]. The mitochondrial calcium uniporter is responsible for fast and electrogenic Ca^2+^ uptake to mitochondria (calcium is transported against the concentration gradient using the electrochemical gradient—mitochondrial membrane potential) [3,4,5]. Mitochondrial calcium transport is coupled to the accumulation of inorganic phosphate [6,7], and Ca^2+^ and phosphate are stored in form of osmotically inactive precipitates. In the mitochondria of brain cells, Ca^2+^ is stored mostly in bound form with a bound/free ratio after stimulation of ~4000 [8]. Free Ca^2+^ is transported to the cytosol of brain cells by the Ca^2+^/Na^+^ exchanger [9,10], molecularly identified as NCLX (SLC24A6 family) [11] (Figure 1). The balance of the mitochondrial Ca^2+^ is vitally important because an overload of calcium in the matrix of mitochondria leads to the opening of the mitochondrial permeability transition pore (mPTP). Several possible structures of the mPTP with various protein compositions were suggested previously (including ANT, a dimer of F_0_F_1_-ATPase, or its subunits), and these are currently the subject of intensive discussion. However, as of now, molecular inhibition has confirmed that only a deficiency in the matrix protein cyclophilin D (CypD) leads to the inhibition of the permeability transition [12]. The process of mPTP opening is an initial step in the release of proapoptotic proteins and the processes of necrotic and apoptotic cell death.

Multiple redox processes in cells adapt to reactive oxygen species and the products of oxidation and play a role in signal transduction [13]. Mitochondria play an important role in redox signaling and produce ROS in several enzymes with the major implication of the electron transport chain, which, in contrast to other mitochondrial and nonmitochondrial enzymes, produces ROS constantly [14,15] (Figure 1). The mitochondrial electron transport chain produces ROS as by-products. This is a consequence of the leak of electrons and the production of ROS (mainly superoxide anion and hydrogen peroxide), which is dependent on the metabolic state, mitochondrial membrane potential etc. Proteins of the mitochondrial matrix—such as the rate-limiting enzymes of the TCA cycle, α-ketoglutarate dehydrogenase or pyruvate dehydrogenase complexes—produce superoxide or hydrogen peroxide independently of the electron transport chain [16] (Figure 1). Importantly, ROS in mitochondria can be produced in enzymes which are not involved in the process of energy production, including those located on the outer mitochondrial membrane, monoamine oxidase (MAO) and cytochrome b5 reductase (Cb5R); enzymes of the inner mitochondrial membrane, such as glycerol-3-phosphate dehydrogenase; and in some cell types, various cytochrome P450 monooxygenases [15]. Importantly, mitochondria are not only producers of ROS but are also targets. Thus, the overproduction of ROS is shown to be an inductor of mPTP opening, and calcium and ROS are known to be one of the major triggers for cell death [17,18].

Mitochondrial and cytosolic ROS and Ca^2+^ interact, complement, and regulate each other physiologically and pathologically.

ROS can stimulate calcium signaling in neurons and astrocytes. The application of catecholamines (adrenaline or dopamine) to astrocytes induces the production of hydrogen peroxide in monoamine oxidase and stimulates lipid peroxidation and the activation of phospholipase C and IP3-induced calcium signaling [19,20]. Mitochondrial ROS under hypoxic conditions in astrocytes play a signaling role to trigger calcium signaling and a physiological response to hypoxia [21]. Free radicals can modify Ca^2+^ channel activity, which also plays the role of a chemosensor [22].

Ca^2+^ stimulates ROS production from different sources; thus, calcium signaling can trigger the activation of the NADPH oxidases expressed in neurons and astrocytes—NOX2 and NOX4 [23,24]. Mitochondrial calcium uptake induces the activation of dehydrogenases and mitochondrial respiration and the hyperpolarisation of mitochondria [25]. An increase in the mitochondrial membrane potential leads to a higher probability of electron leaks and ROS production in mitochondria [26].

Mitochondrial ROS and calcium have complementary effects on the activation of pathological mechanisms in brain cells. Thus, the high capacity of brain mitochondria to produce complexes of Ca^2+^ with inorganic phosphate can protect abiding neurons against calcium-induced mPTP opening and cell death. However, ROS overproduction in neurons and astrocytes can serve as an additional trigger for pathology via a decrease in the threshold for pore opening.

Mitochondrial calcium overload and ROS overproduction also work complementarily to induce DNA damage and the activation of poly(ADP-ribose) polymerase (PARP)—a family of proteins best known to facilitate DNA base excision repair [27,28,29], which leads to the production of PAR, energy deprivation, and cell death via a specific mechanism (parthanatos).

Neurodegenerative disorders are devastating and uncurable diseases. Despite the significant progress in the understanding of the mechanisms of their pathology in recent decades, the initial triggers of these diseases remain unclear. Although in all of these diseases damage to a different brain area is represented, they all share several pathological features, including the aggregation of misfolded proteins, mitochondrial disfunction, and oxidative stress [30,31].

## 2. Huntington’s Disease

Huntington’s disease (HD) is an autosomal dominant neurodegenerative disease and the most common cause of hereditary chorea. The mutation that leads to the development of HD is the expansion of CAG repeats (encoding glutamine) in exon 1 of the HTT gene, which is responsible for the synthesis of the huntingtin protein [32]. Huntington’s disease is characterized by a great phenotypic variety of motor, cognitive, and mental disorders. There is a correlation between the number of CAG repeats and the age of HD debut, as well as the severity of manifestations of the disease. As a result of a specific mutation, the mutant huntingtin protein (mHtt) is able to initiate a cascade of damage to molecular processes, ultimately leading to mitochondrial dysfunction, ROS formation, and increased oxidative stress [33,34,35,36]. Mutated huntingtin is shown to be associated with mitochondria, which can explain the effect of this protein on mitochondrial function [37].

The mitochondria of the striatum are the site of the integration of multiple pathological stimuli, including calcium deregulation and ROS production, which lead to the opening of the mPTP followed by a cascade of subsequent events of cell death [30]. Metabolic and mitochondrial dysfunction in HD results from the deregulation of the mitochondrial calcium efflux that alters intracellular Ca^2+^ homeostasis [38]. It should be noted that disturbances in intracellular Ca^2+^ homeostasis have been reported possibly in all experimental HD models [39,40].

CAG repeat in the huntingtin gene has been shown to induce the overactivation of the NMDA receptor, which leads to an increased calcium influx, profound mitochondrial depolarization, a decrease in ATP synthesis, and excitotoxicity. These conditions are also associated with the activation of NADPH oxidase and ROS production [41,42,43,44].

Cytoplasmic Ca^2+^ overload in striatal neurons can lead to the increased production of ROS through the mitochondrial production of superoxide, resulting in cell death [45]. Results obtained using HD mouse model neurons and HD patient fibroblast cells showed that excessive mitochondrial oxidative stress is critically dependent on mitochondrial Ca^2+^ loading in HD cells. The blockade of mitochondrial Ca^2+^ uptake abolishes the increased superoxide formation [46]. Moreover, mitochondrial loading with Ca^2+^ in HD cells caused a 2× higher level of mitochondrial genomic DNA damage due to excessive mitochondrial ROS production.

Mitochondria isolated from cells obtained from both HD patients and HD mouse models are more sensitive to Ca^2+^ loading, i.e., they have a significantly lower threshold required to trigger mPTP opening [47,48,49,50,51].

The decreased mitochondrial calcium threshold in HD mitochondria can be associated with the ability of huntingtin to directly produce free radicals [52].

It has been shown that while mitochondria are unable to withstand high Ca^2+^ loads [53], there is an increased transcription of genes involved in ROS cleavage (Gpx1, catalase, SOD1, and SOD2), indicating that the relation between Ca^2+^ and ROS is the most likely inducer of mitochondrial stress in HD.

It is important to note that other researchers have failed to detect a defect in Ca^2+^ accumulation in mitochondria exposed to mHtt with an elongated polyQ site [54,55,56]. Differences in the data may be related to the specific model or experimental study protocols in question, as well as the need to consider the role of other mechanisms in HD pathogenesis, such as changes in mitochondrial dynamics [57].

Impaired mitochondrial function and Ca^2+^ homeostasis can be explained by changes in mHtt-induced division regulation. Aberrant Drp1-mediated mitochondrial fragmentation causes mitochondria to move away from the endoplasmic reticulum. The loss of ER–mitochondrial contacts leads to excessive mitochondrial ROS (superoxide) production and decreased mitochondrial Ca^2+^ uptake, causing an aberrant increase in cytosolic Ca^2+^ and, consequently, altered calcium homeostasis [58]. Drp1-mediated mitochondrial fragmentation and increased propensity for apoptosis in HD cells were also observed in [59].

Although the role of the mitochondrial calcium deregulation in HD is proven by a number of publications and there are no doubts about the role of ROS in the pathogenesis of this disease, the nature of the interaction between these processes in the mechanism of neurodegeneration is not clear.

## 3. Alzheimer’s Disease

Alzheimer’s disease (AD) is the most common neurodegenerative disorder and is characterized by a very high incidence among the elderly. The presence of mutations in three alternative genes, APP, PSEN1 and PSEN2, and the absence of any specific genetic lesions makes it possible to distinguish between familial and sporadic forms of this disease [60].

The major histopathological features of this disease, first described more than 100 years ago, are extracellular senile plaques (mostly formed by aggregated β-amyloid (Aβ)), intracellular neurofibrillary tangles (formed by tau protein), and the loss of basal forebrain cholinergic neurons [61]. Major studies into Alzheimer’s disease are focused on unravelling the effects of different forms of β-amyloid, hyperphosphorylated, and/or aggregated tau; on transgenic animals with mutations in APP, PSEN1, PSEN2; or on some genes which increase genetical risk of the disease, such as APOE.

**Figure 2:**
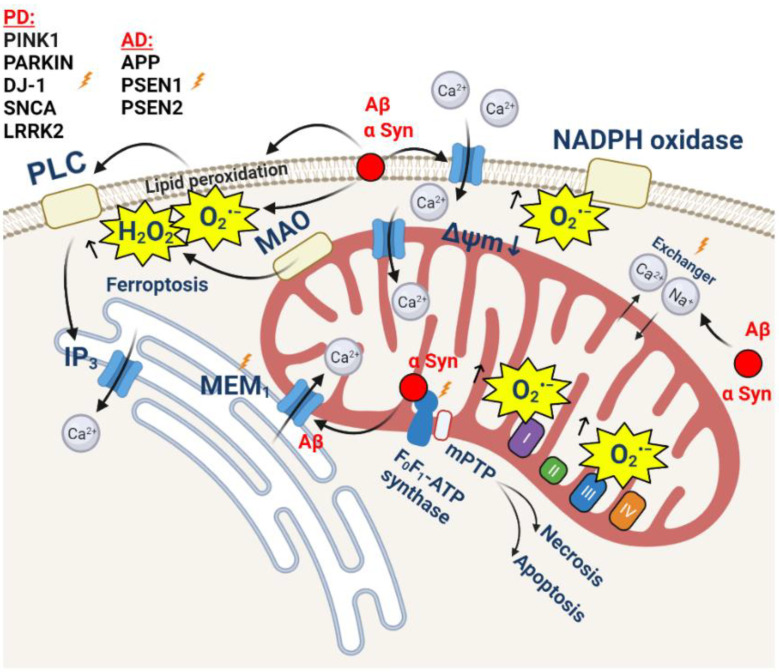
ROS and Ca^2+^ in abnormal calcium signaling leading to cell death in Alzheimer’s disease and Parkinson’s disease. Aggregated βAmyloid or α-synucleins form calcium-permeable channels on plasma membrane. PD and AD mutations lead to inhibition of NCLX, massive ROS production, and calcium overload in mitochondria.

β-amyloid is able to produce channels on the membranes [62] that induce calcium signals in astrocytes but not in neurons, due to the higher content of cholesterol [63,64]. Importantly, oligomeric Aβ can induce calcium signals in picomolar concentrations [65] and form pores in the membranes of lysosomes [66]. High calcium signaling induces the activation of NAPH oxidase; mitochondrial calcium uptake [2,67]; and the combination of ROS and calcium in mitochondria, which induces the activation of PARP and mPTP opening [68] (Figure 2). The importance of calcium overload was also demonstrated in transgenic models of AD. Thus, in an APP/PS1 Tg mouse model of cerebral β-amyloidosis, Aβ accumulation provoked mitochondrial Ca^2+^ overload, leading to neuronal death. The inhibition of the MCU by the specific channel blocker Ru360 abolished mitochondrial Ca^2+^ uptake and was protective [69]. The importance of mitochondrial calcium overload and ROS in the pathology of AD was also proven in different mouse models of the disease (which developed both amyloid plaques and neurofibrillary tangles), showing that the loss of NCLX expression and functionality led to the progression of AD by promoting superoxide generation, mitochondrial calcium overload, and neuronal cell death [70]. However, mitochondrial calcium overload and ROS production via the inhibition of NCLX can be induced by the tau protein [71,72] (Figure 2).

Interestingly, similar to β-amyloid, presenilins (PS1-M146V and PS2-N141I FAD) can form low-conductance divalent-cation-permeable ion channels in ER that selectively release Ca^2+^ [73]. This increased intracellular Ca^2+^ and led to mitochondrial dysfunction and apoptosis. However, these results are still disputable and have not been confirmed by other groups [74,75,76].

ER–mitochondrial contacts are directly associated with mitochondrial calcium uptake. Thus, Aβ initiates Ca^2+^ release from the ER via IP3Rs or RyRs. Mitochondrial Ca^2+^ overload can be the result of an increase in ER–mitochondria coupling and the transfer of Ca^2+^ from the ER to mitochondria. This has been shown in studies on fibroblasts from AD patients and a neuroblastoma cell line containing a familial presenilin-2 (PS2) AD mutation [77].

The importance of mitochondrial calcium overload in combination with excessive ROS production leading to mPTP opening and cell death was proven in the studies with cyclophilin-D-deficient mice, which exhibited the absence of cognitive decline [78,79] (Figure 2).

The model of Aβ42 oligomer-mediated oxidative damage and subsequent neurotoxicity in AD in the work of Butterfield and Halliwell demonstrates the deregulation of calcium homeostasis in this disorder [80]. Studies of an AD brain identified indications of oxidative damage due to the effects of Aβ oligomers [81]. This damage follows the path of lipid peroxidation, the major and highly reactive product of which is protein-bound 4-hydroxy-2-trans-nonenal. Oxidative dysfunction would have the effect of decreasing the neuronal cell potential and thus opening voltage-gated Ca^2+^ channels (VGCCs). The opening of VGCCs leads to a massive increase in intracellular Ca^2+^ due to the difference in the calcium concentration inside and outside the cell. This can overwhelm intracellular Ca^2+^ stores in the ER and mitochondria. In the case of mitochondria, elevated mitochondrial Ca^2+^ can lead to the opening of the mitochondrial permeability transition pore, with the subsequent release of cytochrome C and induction of apoptosis [82].

## 4. Parkinson’s Disease

Parkinson’s disease (PD) is a neurodegenerative pathology associated with the death of dopaminergic neurons in substantia nigra. In the same way as Alzheimer’s disease, the histopathological features of PD are intracellular inclusions—Lewy bodies, which mainly consist of aggregated α-synuclein. Mitochondria have been associated with PD ever since the discovery was made that mitochondrial toxins such as rotenone and 1-methyl-4-phenyl-1,2,3,6-tetrahydropyridine (MPTP) cause the death of dopaminergic neurons and permanent symptoms of Parkinson’s disease in cellular and animal models [83,84,85,86]. Moreover, the discovery of familial forms of PD confirmed the strong association of PD pathology with mitochondria. Thus, mutations in PINK1 and PARKIN (which encode proteins involved in mitophagy), DJ-1 (which encodes redox-sensitive chaperone deglycase DJ-1, inhibiting the aggregation of α-synuclein during oxidative stress), SNCA (which encodes α-synuclein), and LRRK2 (which encodes leucine-rich repeat kinase 2) were all proven to cause the development of PD. The products of these genes have constant or partial co-localization with mitochondria and the dysfunction of these proteins causes mitochondrial damage through high levels of oxidative stress, mitochondrial calcium, and energy deficit [87].

A vast amount of the studies which report on the activity of α-synuclein in various processes are mainly focused on the aggregated toxic proteins. However, monomeric α-synuclein is involved in synaptic transmission [88,89] and can induce calcium signaling in neurons and astrocytes [90] (Figure 2). Recently, we demonstrated that monomeric α-synuclein can directly interact with mitochondrial F_0_F_1_ ATP synthase and modulate its activity [91]. Oligomeric α-synuclein can also induce calcium signaling in neurons and astrocytes and stimulate ROS production, partially by activating ROS-producing enzymes (including mitochondrial ROS), but mainly by generating superoxide by itself in interaction with metal ions [92]. Mitochondrial Ca^2+^ uptake, together with the binding of oligomeric α-synuclein to the same site of mitochondrial F_0_F_1_ ATP synthase where monomers bind, produces oxidation of this complex and induces mPTP opening and cell death [93] (Figure 2). Interestingly, the direct contact of oligomeric α-synuclein with lipids from membranes or other intracellular organelles leads to a profound lipid peroxidation, calcium deregulation, and ferroptosis [94,95].

Additionally to redox changes, oligomeric α-synuclein also the inhibits mitochondrial Na^+^/Ca^2+^ (NCLX) exchanger and leads to calcium overload in the matrix of mitochondria [96]. Importantly, the α-synuclein-associated mutation A53T has been shown to inhibit NCX3 (the plasmalemmal Na^+^/Ca^2+^ exchanger which is shown to be located on the mitochondrial membranes as well) and induce mitochondrial disfunction and the loss of striatal neurons [97]. The inhibition of NCLX has been shown for familial forms of PD and was identified for first time in PINK1 deficiency and mutations [98,99], and later in LRRK2 deficiency and overexpression [100,101]. The restoration of mitochondrial Ca^2+^ in models with an overexpression of NCLX or PINK1 has been shown to be protective for PINK1-deficient neurons, LRRK2, and cardiomyocytes in sepsis [100,102,103] (Figure 2). The protection of neurons with inhibited NCLX can also be achieved by the molecular inhibition of the MCU or by the sequestration of calcium uptake by pharmacological compounds [104,105,106]. In contrast, the inhibition of NCLX by CGP-37157 is shown to be protective against α-synuclein- and rotenone-induced cell death [107], which can be explained by its differences from the calcium overload mechanism, including the ability of CGP-37157 to inhibit some other proteins, such as L-type voltage-dependent Ca^2+^ channels [108]. Considering their low specificity, some forms of calcium antagonists may have limited therapeutic value in PD.

However, mitochondrial calcium overload is not the sole activator of neuronal cell death. Thus, PINK1-deficient neurons induce excessive ROS production in NADPH oxidase and mitochondria, and the additional ROS produced in MAO under the application of dopamine induce mPTP opening and cell death [109]. 

The overproduction of ROS and mitochondrial calcium overload are also responsible for the activation of poly(ADP-ribose) polymerase-1 (PARP-1), the induction of PAR, and the triggering of cell death. There is a growing body of evidence for the involvement of this pathway in the etiopathology of PD. Thus, the interaction of PAR with phosphorylated α-synuclein was found in post mortem PD samples and a murine model of α-synucleinopathy (M83-SNCA × A53T) [110]. Toxic forms of α-synuclein directly activate PARP-1, and PAR generation accelerates the formation of pathologic α-synuclein, resulting in cell death via parthanatos [111]. The role of PARP-1 activation in the limitation of mitochondrial substrates and the induction of neurodegeneration was also found in models of familial forms of PD—Fbxo7 [112] and PINK1, where the supplementation of cells with NAD+ was shown to be protective [113]. A PARP-1 mutation has been shown to be protective against mitochondrial dysfunction and neurodegeneration in a PARKIN model of Parkinson’s disease, which also confirms the importance of PARP-1 activation in the mechanism of the neurodegeneration of PD and the role of mitochondrial processes in this activation [114].

## 5. Frontotemporal Dementia

The term frontotemporal dementia (FTD) refers to a group of neurodegenerative diseases characterized by the degeneration of the frontal and temporal lobes of the brain with an early onset and associated with disorders in behavior, language, and cognition [115,116]. More than 20% of FTD cases belong to familial forms. The mutations of the MAPT gene, which encode the microtubule-associated protein TAU, are the most common mutations associated with FTD [117]. The involvement of the tau protein in the pathogenesis of FTD brings this disorder close to tauopathies—a group of diseases characterized by an abnormal tau profile [118]. However, there are number of genes, including the TDP-43 and FUS proteins, which induce FTD but are also involved in amyotrophic lateral sclerosis. Thus, the loss of function of TDP-43 impairs mitochondria–ER contacts, which induces the alteration of mitochondrial calcium uptake [119].

The cellular metabolism imbalance observed in FTD may be associated with the deregulation of mitochondrial calcium homeostasis and ROS production. Thus, changes in ER–mitochondrial contacts is one of the features of both FTD and ALS, and changes in these contacts may lead to the changes in mitochondrial Ca^2+^ [120,121,122] in FTD-associated FUS, TDP-43, and C9orf72.

Aggregates of the recombinant TAU protein P301S can also form a channel in the same way as β-amyloid or α-synuclein [123,124]. However, aggregated tau induces calcium signaling not through these channels but via the activation of voltage-gated Ca^2+^ channels, and activates ROS production in NADPH oxidase [123]. In a human neuronal FTD model with 10 + 16 mutation of MAPT [125], a significant increase in the mitochondrial membrane potential due to the complex V reverse activation was found. As a result, an increase in ROS production, leading to the development of oxidative stress and cell death through the opening of mPTP was shown [125]. On the other hand, excessive mitochondrial ROS production in these neurons damages the proteins responsible for the trafficking of AMPA and NMDA receptors, which leads to changes in calcium signaling and the electric activity of FTD neurons [126,127]. Importantly, neuronal activity in these cells and glutamate-induced calcium signaling was effectively restored by the incubation of these cells with mitochondrial antioxidants but not with general antioxidants or PUFAs. Interestingly, ROS and changes in mitochondrial calcium handling are shown to be responsible for the changes in the expression and function of the glutamate receptors AMPA and NMDA and the corresponding increase in the vulnerability of neurons to excitotoxicity [126,128,129]. 

It should be noted that the tau protein with the same mutation that leads to FTD induces the inhibition of NCLX in primary neurons and astrocytes, leading to mitochondrial calcium overload and, in combination with high ROS production, inducing mPTP opening, which can explain the toxicity of this form of tau (K18) [72].

## 6. Amyotrophic Lateral Sclerosis

Amyotrophic lateral sclerosis (ALS) is a rapidly progressing neurodegenerative disease with a predominant degradation of motor neurons and skeletal muscle dystrophy [130]. The most common cause of death, usually within 2–5 years of diagnosis, is respiratory failure [131,132]. About 10% of reported ALS cases are associated with mutations in more than 20 genes, including the genes of Cu/Zn superoxide dismutase (SOD1), fused in sarcoma (FUS), and alsin (Als2) [133,134]. However, despite the different etiology, mitochondrial dysfunction is one of hallmarks of its pathology [135]. Morphological changes (including swelling and fragmentation), an ATP production decrease, and electron transport chain failure was detected by different research groups [136,137,138,139]. Mitochondria play an important role in the closely related processes of calcium homeostasis and the production of reactive oxygen species (ROS). The dysregulation of these processes is one of the mechanisms of ALS development [140,141].

Oxidative damage is one of the most important causes of the degradation of both motor neurons and skeletal muscles. Motor neurons have a higher sensitivity to increased ROS production, which was proven in a number of studies. Thus, excessive ROS production leads to excitotoxicity in motor neurons [132,133,134,135,136,137,138,139,140,141,142]. This can be explained by higher levels of the expression of AMPA-receptors with high calcium permeability, due to a lower content of the GluR2 domain [143,144,145] or due to a mutant SOD1 [146]; it can also be explained by the low expression of calcium-binding cytoplasmic proteins, such as parvalbumin and calbindin D-28k, which leads to the 5-6-fold reduced capacity of the cytosol for calcium [147,148,149,150]. Importantly, it can be due to a reduced physiological mitochondrial capacity for calcium, and in the case of the mouse mutant SOD1 model, G93A is shown long before the manifestation of the pathology phenotype. This may be due to the reduced density of the mitochondrial network compared to nonmotor neurons [151,152]. Such a combination of reasons explains the degradation of motor neurons in conditions where other cells retain their functionality [153,154].

**Figure 3:**
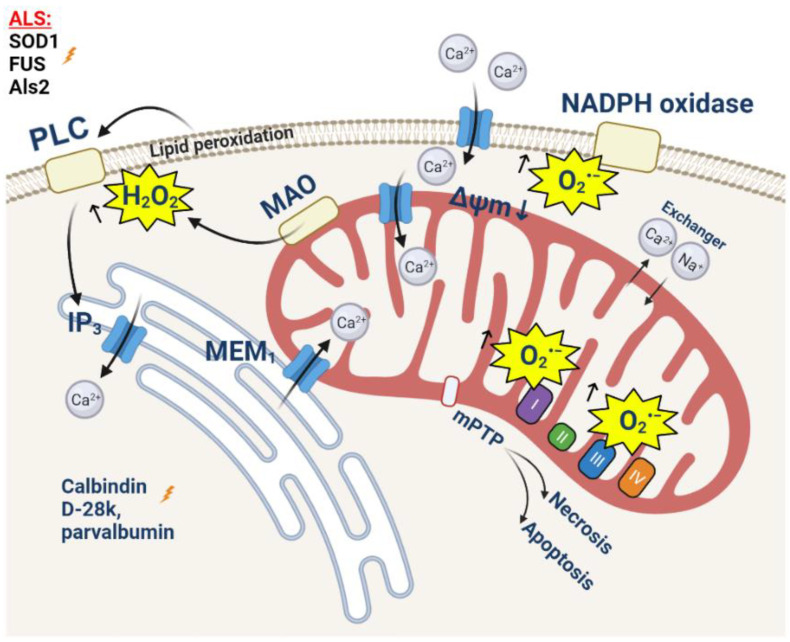
Amyotrophic lateral sclerosis: ROS and calcium in neurons. ALS-associated mutations induce mitochondrial dysfunction and decrease antioxidant defense in mitochondria, altering the MEM and mitochondrial calcium handling.

After the stimulation of AMPA-receptors with glutamate, the concentration of cytoplasmic calcium increases dramatically, which can lead to delayed calcium deregulation and cell death [155]. The transport of calcium into intracellular depots, mainly into the endoplasmic reticulum (ER) and mitochondria, is one of the ways to reduce cytoplasmic calcium. Taking into account the low level of calcium-binding proteins, the loading of these organelles with Ca^2+^ increases significantly. A number of studies prove (using mutant SOD1, Sig1R, TDP-43, and FUS) a significant degradation of mitochondria-associated membranes, which play an important role in mitochondrial calcium homeostasis [120,156,157,158] (Figure 3). However, due to a reduced capacity, the mitochondria of motor neurons reach the calcium threshold much faster compared to control cells. This results in a significant decrease in the mitochondrial membrane potential [159] (Figure 3), and an increase in the rate of the production of mitochondrial ROS and the opening of the mitochondrial permeability transition pore [160] (Figure 3). The induction of oxidative stress affects the surrounding astrocytes, in particular, by reducing the activity of glutamate transporters [154]. Thus, there is a vicious cycle of constant increases in the level of oxidative stress in motor neurons and surrounding astrocytes [161].

The mechanism of motor neuron degradation mediated by calcium homeostasis disorders and ROS overproduction is shown in the non-cell-autonomous model of ALS [131]. In this experiment, WT motor neurons were cultivated in astrocyte-conditioned media (ACM) containing toxic products of astrocytes expressing SOD1G93A. An increase in the excitability of neurons due to a higher level of Nav-channel permeability and an increase in the concentration of calcium in the cytosol in mitochondria was shown [131]. This led to a decrease in the mitochondrial membrane potential and an increase in ROS production and the opening of the mPTP. The overproduction of ROS also leads to the phosphorylation of apoptosis-inducing tyrosine kinase (cAbl) [131]. As a result, approximately 50% of motor neurons die within a few days [162,163,164].

The muscular dystrophy observed in ALS is not solely a consequence of the motor neuron degradation, but can also be explained by additional mechanisms, with the leading role played by calcium homeostasis disorders and oxidative stress [165,166,167]. The primary changes are observed in the neuromuscular junctions (NMJ), which, in particular, is shown in the cells of the SOD1G93A model [168]. In muscle fibers, isolated at the age of ALS onset, a significant drop in the mitochondrial membrane potential was shown as a result of an increase in intracellular calcium transient [166,169]. An approximately 14.6% decrease in the calcium uptake of depolarized mitochondria was shown while cytosolic calcium was increased in these cells by 15.6% [170]. This activates the aggregation of mutant proteins (in particular SOD1) in mitochondria and the decrease in the mitochondrial membrane potential, mitochondrial calcium uptake, and activation of ROS production in both mitochondria and the cytosol [135,166,171]. On the other hand, prolonged muscle denervation can lead to an increase in the basal concentration of calcium in the cytosol followed by a mitochondrial calcium overload and ROS overproduction.

## 7. Conclusions

The similarity of the mechanisms of neurodegeneration of most common neurodegenerative disorders and the involvement in these processes of mitochondrial calcium and ROS production raise the question of whether the path to neuronal cell death is universal; why is neuronal loss so specific to brain regions in various diseases—from the frontotemporal area and cortex to the striatum or motor neurons? This may be explained by the differences in the rates of brain energy metabolism [172], the variability in basal reactive oxygen species production and the level of antioxidants in different brain areas [173], or the variations in neuronal activity [174]. One of the possible explanations could be the specificity of misfolded proteins to certain areas or neuron–glia interaction [175]. It should be noted that the term ROS is too broad and encompasses the toxic species (hydroxyl radical and superoxide anion), while hydrogen peroxide and singlet oxygen may have a positive and even protective effect on neurons [176]. ROS and calcium in mitochondria are contributors to mPTP opening—the final step before the induction of cell death, a mechanism that is universal for all cell types.
